# Insular gliomas and tractographic visualization of the connectome

**DOI:** 10.3171/2021.10.FOCVID21194

**Published:** 2022-01-01

**Authors:** Nicholas B. Dadario, Charles Teo, Michael E. Sughrue

**Affiliations:** 1 Robert Wood Johnson Medical School, Rutgers University, New Brunswick, New Jersey;; 2 Centre for Minimally Invasive Neurosurgery, Prince of Wales Private Hospital, Randwick, New South Wales; and; 3 Omniscient Neurotechnology, Sydney, New South Wales, Australia

**Keywords:** connectome, glioma, insula, salience network, tractography, artificial intelligence, language, central executive network

## Abstract

In this video, the authors present a connectome-guided surgical resection of an insular glioma in a 39-year-old woman. Preoperative study with constrained spherical deconvolution (CSD)–based tractography revealed the surrounding brain connectome architecture around the tumor relevant for safe surgical resection. Connectomic information provided detailed maps of the surrounding language and salience networks, including eloquent white matter fibers and cortical regions, which were visualized intraoperatively with image guidance and artificial intelligence (AI)–based brain mapping software. Microsurgical dissection is presented with detailed discussion of the safe boundaries and angles of resection when entering the insular operculum defined by connectomic information.

The video can be found here: https://stream.cadmore.media/r10.3171/2021.10.FOCVID21194

## Transcript

In this video, we demonstrate the use of connectomics and tractographic visualization using artificial intelligence to help improve the resection of an insular glioma. So reviewing the anatomy of the large-scale brain networks.

### 0:33 Default Mode Network.

First, we will start with the default mode network.^1^ The default mode network, or DMN, is an anterior cingulate, posterior cingulate, and lateral parietal network. You can see that the two medial components, the anterior cingulate and posterior cingulate components, are connected via the cingulum bundle, with the lateral parietal component being an isolated island at least relevant to these networks. The default mode network does a variety of functions. It is critical to cognition and emotional regulation, most notably episodic memory, theory of mind, and imaginative thinking.

### 1:11 Salience Network.

The anatomy of the salience network is shown here. This is a middle cingulate and anterior insula network. You can see that the two components are connected via the frontal aslant tract, and the frontal aslant tract is named because it runs aslant to the overall anterior-posterior white matter of this part of the brain.^2^ If you look at the middle cingulate component, it is important to note that in addition to the areas that are in the Brodmann area 32 complex, as you can see in the upper left, there is an area called SCEF, which stands for supplementary and cingulate eye field. This area is better thought of as a key component of both the salience network and the supplementary motor area. Importantly, a good portion of the supplementary motor area is in fact subcomponents of the default mode network and salience network.

### 2:15 Central Executive Network.

Finally, the central executive network is best thought of as the opposite network to the DMN. So where the DMN is handling internal mental processes, the central executive network is handling active thinking and active processes. It has a four-component aspect with a thin portion of the superior longitudinal fasciculus connecting these areas. And this is comprised of the frontal polar region, as seen on the left in the upper left image; an area called 8C, which is one of the most highly connected portions of the human cerebrum; area PFm, which is in the supramarginal gyrus; and area TE1m, which is in temporal lobe in the middle temporal gyrus.

### 3:07 Network Switching.

Now these networks, the central executive network and the default mode network, are generally not firing at the same time. But the salience network mitigates a switch that switches between the internal and external mental world networks, or the DMN and CEN. This highlights the role of the salience network in decision-making and decision-action activation.^3^ More importantly, by understanding the interplay of these axes, we can begin to grasp a better understanding of how to avoid complications when working around these networks.^4,5^ In this particular case, both the central executive network and salience network have components that are around the insula and thus are at risk from this operation.

### 3:55 Clinical Presentation.

Patient is a 39-year-old woman who presents with a very complex history. She initially had symptoms of a somatization disorder and depression, but later on developed a seizure and sensory disturbances. The depression is relevant in this case, as we will show in a bit. She eventually was found to have a low-grade glioma, which was centered mostly in the left insula, the superior temporal gyrus and operculum, and the temporal stem and the base of the amygdala and hippocampus.

### 4:21 Preoperative Planning With Tractography.

The particular question we had in this case was multifold. As you can see in this image in the Quicktome software, the most common concern that people have is ultimately the corticospinal tract. In this case, its usual with insular gliomas that this is mostly medial to the tumor, but it’s important to note if you exit the superior deep boundary of an insular glioma, you can hit it.

### 4:48 Identifying Relevant Networks and Tracts.

Interestingly, when we look at the IFOF segmentation, and we have included the normal IFOF for comparison, you can see that the inferior fronto-occipital fasciculus is missing on the side of the tumor. When we look at the objects regarding the salience network, which include the frontal aslant tract (FAT) as well as the nodes of the salience network seen here in the 3-dimensional image, you will note that the salience network has been pushed upward by the tumor and distorted. Given its role in depression as well as being aware of your interoceptive sensations, this network disruption makes the likely cause of her symptoms.

### 5:24 Language System.

Now what we are trying to determine with the language system of course is how far back can we cut the superior temporal gyrus safely. As you can see, the majority of this tumor is safe, but the cut on the superior temporal gyrus has to be posteriorly angled. You can see here on the sagittal cut that, again, we have a fairly clear angle into the tumor.

### 5:47 Exposing the Insula.

Now you can see here, in the intraoperative setting, we have the temporal lobe, and what we are first going to start off with is making a cut from anterior-posterior to begin to work inside this keyhole craniotomy. You can see we can utilize the image guidance with the Quicktome outputs to visualize these networks. This allows us to first cut and remove the majority of the temporal pole underneath the bone flap with a cut that safely works along the STG to expose the insula. Now notice that we again have respected the anterior boundary of the temporal portion of the language system as seen in this combined shot of the image guidance with the Quicktome output and the brain tumor.

### 6:35 Insular Resection.

You can see that now we have identified the temporal stem. You can see that where the image guidance is helpful here is making that cut in an angle that preserves the connectivity of the language system. The insular resection is mostly an anatomical resection, as we know that the IFOF as well as the salience network are not located within the parenchyma within this tumor. And again, we define windows and remove circumflex arteries to begin debulking the insula. But the goal is making the insula plane aligned with the hippocampus and amygdala. Once we have done that, we begin seeing the characteristic appearance of the basal ganglia, which we know as our deep boundary.

### 7:23 Angled Resections.

Again, at this point, we are attempting to work posteriorly along the superior temporal operculum. As you can see, this tumor, as seen at about 5 o’clock on this diagram, is tucked underneath the language system. We know that we cannot work more posteriorly; we must work underneath that overlying cleft, because otherwise we would be violating the language system. This makes this angle more difficult, but ultimately what is necessary to achieve a good speech outcome for this patient.

### 7:58 Debulking the STG.

As you can see, we are taking a steep posterior angle, identifying the posterior insular vessels, and then using this to safely debulk the superior temporal gyrus component under the language system. Now if the Quicktome output had told us that we could remove some overlying cortex, this of course would have made this somewhat simpler.

### 8:20 Superior Boundary.

We are now working on the superior boundary, and you can see portions of the lenticulostriate arteries and basal ganglia. Again, you could see the angle that we did to safely enter this very difficult portion of the insula through this transoperculum approach. You can also see, as we saw in the preoperative images, its close proximity to the corticospinal tract.

### 8:48 Identifying Basal Ganglia.

While image guidance is helpful for identifying basal ganglia tissue, another helpful guidepost to know how deep you can go is once you begin to identify white speckles, you’re in the basal ganglia.

### 9:04 Superior Portion of Insula.

Now the superior part of the insula is the most dangerous part—not only because it’s a difficult angle. As you can see, we have changed the angle of the microscope and we are working between this Y-shaped vessel that we often see within the superior division of the MCA. But we also know that portions of the salience network and corticospinal tract are at risk from the preoperative planning.

### 9:28 Motor and Language Fibers.

So you can see, these are highlighted in blue and red, the blue being motor and the red being language. This shows the exact position of the face motor fibers which are visualizable with constrained spherical deconvolution tractography, which is crossing fiber tractography.

### 9:50 Postoperative Outcome.

So as you can see, an excellent resection of this tumor was performed. Ultimately, the patient had an excellent speech outcome with a gross-total resection of this complex lesion.

### 10:04 Conclusion.

Ultimately, what connectomics provided here was a detailed map of the language system, including cortical regions, which helped us define the safe boundaries and angles of resection and entry into the insular operculum region.

## Disclosures

Dr. Sughrue is an employee of and shareholder in Omniscient Neurotechnology. Dr. Teo is a shareholder in Omniscient Neurotechnology and a consultant for Aesculap. Quicktome is a commercially available product that Omniscient Neurotechnology sells.

## Author Contributions

Primary surgeon: Sughrue, Teo. Editing and drafting the video and abstract: Dadario. Critically revising the work: Sughrue, Dadario. Reviewed submitted version of the work: Sughrue, Dadario. Approved the final version of the work on behalf of all authors: Sughrue. Supervision: Teo.
